# Autophagy-related biomarkers in preeclampsia: the underlying mechanism, correlation to the immune microenvironment and drug screening

**DOI:** 10.1186/s12884-023-06211-2

**Published:** 2024-01-02

**Authors:** Rui Wan, Peng Yao, Yuxiu Wang, Lele Zhang, Wei Guo, Meiying Du, Yu Wang, Wenbiao Shi, Weili Li

**Affiliations:** 1 Department of Obstetrics, Huanghe Sanmenxia Hospital Affiliated to Henan University of Science and Technology, Sanmenxia, China; 2 Department of Clinical Laboratory, Huanghe Sanmenxia Hospital Affiliated to Henan University of Science and Technology, Sanmenxia, China; 3 Department of Gynecology and Obstetrics, The First Affiliated Hospital of Shandong First Medical University & Shandong Provincial Qianfoshan Hospital, Jinan, China; 4 Health Examination Center, Shanghai Children’s Medical Center Hainan Branch, Shanghai Jiao Tong University School of Medicine, Sanya, China; 5 College of Chemical Engineering, Shijiazhuang University, Shijiazhuang, China; 6 Department of Obstetrics, Qujing Maternal and Child Health Hospital, Qujing, China; 7 Zhangjiang Center for Translational Medicine, Shanghai Biotecan Pharmaceuticals Co., Ltd, Shanghai, China

**Keywords:** Pre-Eclampsia, Autophagy, Biomarkers, Cellular microenvironment, Molecular docking simulation

## Abstract

**Background:**

Preeclampsia is a life-threatening disease of pregnancy that lacks effective pharmaceuticals which can target its pathogenesis. Since preeclampsia involves complex pathological processes, including autophagy, this study aims to explore autophagy-related mechanisms of preeclampsia and to screen potential drugs.

**Methods:**

Firstly, the datasets GSE75010, GSE24129, GSE66273, and autophagic genes lists were downloaded from public databases. Then, a weighted gene co-expression network analysis (WGCNA) was applied to filter autophagic-related hub genes of preeclampsia. The differential expression levels of the hub genes were validated with datasets GSE24129 and GSE66273. Next, the GO and KEGG enrichment, protein-protein interacting (PPI) network, as well as the downstream pathways was analyzed via the starBase, STRING and Cytoscape to determine the functions and regulatory network of the hub genes. Additionally, the immune microenvironment of preeclampsia was investigated by the CIBERSORTX database. Finally, three herb ingredients, berberine, baicalein, and luteolin were screened by molecular docking in comparison to pravastatin, metformin, and aspirin, to predict potential drugs for treating preeclampsia.

**Results:**

A total of 54 autophagy-related genes were filtered by WGCNA. After filtering with |GS| > 0.5 and |MM| > 0.8, three hub genes, namely PKM, LEP, and HK2, were identified and validated. Among these genes, PKM and LEP were overexpressed in women older than 35 years old ( *p*<0.05; *p*<0.05); the expression of PKM, LEP, and HK2 differed remarkably in women with different BMI (all *p*<0.05); PKM overexpressed in women with hypertension (*p*<0.05). The regulatory network of hub genes demonstrated that they were mainly enriched in metabolic pathways, including the AMPK signaling pathway, glucagon signaling pathway, adipocytokine signaling pathway, and central carbon metabolism. Then, immune microenvironment analysis turned out that M2 macrophages were reduced in preeclampsia women (*p*<0.0001) and were negatively correlated with the expression of PKM (*r*=-0.2, *p*<0.05), LEP (*r*=-0.4, *p*<0.0001), and HK2 (*r*=-0.3, *p*<0.001). Lastly, molecular docking showed baicalein and luteolin could bind intimately to hub genes.

**Conclusion:**

PKM, LEP, and HK2 could be promising biomarkers for preeclampsia, which might regulate the pathogenesis of preeclampsia via metabolism pathways and immune microenvironment. Baicalein and luteolin could be potential therapeutics for preeclampsia.

**Supplementary Information:**

The online version contains supplementary material available at 10.1186/s12884-023-06211-2.

## Introduction

Preeclampsia (PE) is a multi-system disorder that classically presents with newly onset hypertension after 20 weeks of gestation, complicated by at least one other end-organ dysfunction, such as proteinuria, renal dysfunction, pulmonary edema, and fetal growth restriction [1]. PE infringes on 4.6% of pregnant women worldwide [2, 3] and 2.3% of all pregnancies in China [4], and remains a major cause of maternal and perinatal morbidity and mortality [5]. Regardless of the timing of onset, all PE patients are at risk of rapid deterioration into hemolysis, elevated liver enzymes and low platelet count (HELLP) syndrome or eclampsia [6–8], posing a huge burden to healthcare across the world. Except for these short-term risks, the long-term impact, such as cardiovascular, renal, and cerebrovascular events, posed a threat to both the mothers and their offsprings [9–12]. The etiology of PE remains largely unknown, but placental dysfunction is thought to be the core pathological change for PE. According to the two-stage model, placental dysfunction (stage 1), featured by oxidative stress, mitochondrial dysfunction, metabolism disorder, and apoptosis [13, 14], is the initiating event for the subsequent maternal disease (stage 2) by releasing soluble toxic materials into the maternal circulation [15]. During this process, multiple pathogenic processes, including angiogenic imbalance, immune cell activation, and endothelial cell dysfunction were involved [16–18], but the underlying molecular mechanism remains obscure. Currently, delivery of the fetus is the definite treatment of PE, however, preterm and the long-term risk of complications have brought impediments to PE management. Pharmaceutic therapies have been used in PE management. For example, aspirin is recommended for PE prevention in high-risk pregnant women [19]. Other small molecules, such as statins and metformin, have been shown to reduce adverse outcomes and incidence of PE [20, 21]. However, there is still a great gap between the treatment options and clinical needs, especially in fetal outcomes. Therefore, a comprehensive understanding of pathogenesis and novel agent development will be of capital importance to PE treatment. Autophagy, an intracellular degradation system for damaged or dysfunctional cellular components, plays a capital role in maintaining cellular homeostasis [22]. It is also the core molecular pathway that regulates embryo development during normal pregnancies [23]. Intervention in normal autophagy processes will lead to a plethora pathologies, including cancers, metabolic disorders, and many others [24]. Recently, increasing evidence suggests that autophagy is associated with PE [25–27], however, these studies provided conflicted results. For example, Akaishi et al. [28] reported a decreased level of p62, a substrate of autophagy, indicating activated autophagy in PE placentas. On the contrary, Nakashima et al. [29] observed the accumulation of p62 in placental samples from PE patients, suggesting inhibited autophagy in PE. Such a contrast may be result of a complex regulation network and the microenvironment that autophagy involves in. Therefore, a thorough understanding of correlations between autophagy and PE as well as its microenvironment may lead to finding novel targets for PE and the development of novel therapeutics.

Despite minimal treatment options for PE, development of novel therapies is ongoing, including pravastatin, Metformin, and aspirin. However, due to the high demand for safety profiles in pregnant women, safety equals to or even outweighs effect in drug development for PE. In China, medicinal herbs have been a major treatment option for thousands of years, including the pregnant women. Studies [30–32] accumulated that some active ingredients of herbs, such as berberine, baicalein, and luteolin, can exert a protective effect against PE. For example, berberine (BBR), a major component isolated from *Coptis chinensis*, is active on multiple organs in the human body. Studies showed that BBR can regulate metabolic disorders, reduce hypertension, and alleviate preeclampsia by regulating IL-2/IL-10 balances and inhibiting apoptosis [33, 34]. Baicalein (BCL), a flavonoid extracted from *Scutellaria baicalensis Georgi*, is known for its antitumor effect in various malignancies. It was reported that BCL reduced blood pressure by regulating inflammatory and vascular disease-related factors in hypertensive pregnant rats [35]. Also, BCL was reported to protect liver and kidney function by inhibiting apoptosis in a PE model [36]. As *S. baicalensis Georgi* is one of the herbs that were most frequently prescribed for and used by pregnant women [37], BCL seems to be a potential agent with a good safety profile for PE. In addition, luteolin was found to be a candidate for PE treatment by reducing anti-angiogenic sFlt-1 releasing and promoting vasorelaxation of uterine arteries [38, 39]. However, whether these active ingredients can target autophagy remains obscure. Therefore, this study aims to identify novel autophagy-related targets of PE and eventually find a safe therapeutic for PE by drug screening. First, a weighted gene co-expression network analysis (WGCNA) was applied to filter autophagic hub genes that related with PE. The differential expression levels and regulatory network of hub genes were also validated with two independent datasets and analyzed via specific toolkits, respectively. Associations between the hub genes and the infiltrated immune cells were analyzed to explore the role of autophagy in PE microenvironment. Additionally, as the potential therapeutics for PE, berberine, baicalein, and luteolin were screened by molecular docking in comparison to pravastatin, metformin, and aspirin.

## Methods

### Weighted gene co-expression network analysis (WGCNA) based on autophagic gene set

The dataset GSE75010 was downloaded from the Gene Expression Omnibus datasets (GEO, https://www.ncbi.nlm.nih.gov/geo/), which included 80 placental samples from patients with preeclampsia (PE) and 77 placenta samples from patients without preeclampsia (non-PE). The clinical characteristics of these pregnant women can be found in Table 1. Then, a list of 1334 autophagic genes (Supplementary file 1) was obtained from HADb: Human Autophagy Database (http://www.autophagy.lu/), AUTOPHAGY DATABASE (http://autophagy.info/), HAMdb (http://hamdb.scbdd.com/), and GSEA (http://www.gsea-msigdb.org/gsea/index.jsp). The R package WGCNA software was then used to construct a weighted co-expression network of autophagy-related genes that were differentially expressed in PE patients. During WGCNA analysis, we set the optimal soft threshold to be 8. Next, genes were clustered using average linkage and Pearson’s correlation and a hierarchical clustering tree was constructed using dynamic hybrid cutting, with the minimum number of genes per module being 30. After determining gene modules by the dynamic shear method, the eigengenes of each module were calculated, and then clustered and merged the nearby modules into new modules, with a height equal to 0.25. Finally, we studied the module-trait relationships with Eigengene Networks. All data in this study were obtained from public databases and did not require the institutional review board’s approval.

**Table 1:** Clinical characteristics of pregnant women

	PE group (*n*=80)	Non-PE group (*n*=77)	*p*-value
Age (mean ± SD)	33.19±5.59	33.18±5.59	>0.05
Ethnicity (n, %)			>0.05
Caucasian	43(53.75%)	42(54.55%)	
Asian	11(13.75%)	18(23.38%)	
Black	17(21.25%)	7(9.09%)	
East Indian	5(6.25%)	4(5.19%)	
Other	4(5%)	6(7.79%)	
Blood type (n, %)			>0.05
A	30(37.5%)	18(23.4%)	
B	12(15%)	25(32.5%)	
O	33(41.3%)	30(39.0%)	
AB	4(5%)	4(5.2%)	
Maximum systolic BP^a^ (mmHg), median (range)	167.5 (138-220)	130.0 (97-190)	<0.0001
Maximum diastolic BP (mmHg), median (range)	106.5 (88-140)	81.0 (60-120)	<0.0001
Gestational weeks, median (range)	32 (25-39)	35 (25-41)	<0.01
Miscarriage (n, %)			>0.05
Yes	17(21.3%)	23(29.9%)	
No	63(78.7%)	54(70.1%)	

^a^*BP* Blood pressure

### KEGG pathway and GO functional enrichment analysis

Kyoto Encyclopedia of Genes and Genomes (KEGG) pathway enrichment analysis [40], as well as Gene Ontology (GO) analysis, were performed to reveal the enriched pathways and biological functions of modules using the R package “clusterProfiler”. Then, the ggplot2 package was applied to visualize the enrichment result. GO enrichment included biological process (BP), molecular function (MF) and cellular component (CC).

### Hub genes screening and expression analysis

First, the gene significance (GS) and module membership (MM) were calculated. GS refers to the correlation between gene expression in the blue module and PE, while MM represents the correlation between the blue module and genes. Genes with |GS| > 0.5 and |MM| > 0.8 were selected into gene set 1. Then, a protein-protein interacting (PPI) network of the red module was constructed via the STRING database (https://string-db.org/). The plugin cytohHubba of the Cytoscape software (https://cytoscape.org/) was utilized to calculate the connectivity and selected the top 10 genes into gene set 2. Next, the common genes that overlapped between gene set 1 and gene set 2 were filtered to be hub genes. Finally, the Wilcoxon rank-sum test and Kruskal-Wallis test were used to analyze the expression of the hub gene in various groups and the correlations in between.

### Validation of the hub genes with independent datasets

Two independent datasets, GSE24129 and GSE66273, were downloaded from the GEO database (https://www.ncbi.nlm.nih.gov/geo/). GSE24129 dataset contained 8 PE and 8 non-PE cases, while GSE66273 dataset included 6 PE and 5 non-PE cases. The difference in expression of the hub genes was validated with these two datasets via the Wilcoxon rank-sum test. The threshold was set as the adjusted *P*<0.05.

### Potential molecular mechanisms of hub genes

The specific regulatory mechanisms of hub genes were explored with a multifactor regulatory network analysis. We investigated the interactions between hub genes and between hub genes and autophagic genes. Besides, the R package “clusterProfiler” was applied to analyze the signaling pathway that hub genes and autophagic genes involved. In addition, the networkanalyst database (https://www.networkanalyst.ca/) was used to predict miRNA-hub gene interactions, as well as transcription factors (TF) -hub genes interactions. Moreover, we studied the correlations between lncRNA and hub genes with the help of the starBase database (http://starbase.sysu.edu.cn/) and visualized it with Cytoscape software.

### Infiltrated immune cells in PE patients

Infiltrating immune cells of the GSE75010 dataset were analyzed via the CIBERSORTX platform (https://cibersortx.stanford.edu/). LM22 was selected as signature matrix file and B-mode was enabled for batch correction to obtain the proportion of 22 immune cell types for each sample (Supplementary file 2). Then, the differences in the proportion of immune cells between the PE group and non-PE group were analyzed.

### Drug screening via molecular docking for hub target proteins

The structures of the target protein encoded by hub genes were downloaded from the Protein Data Bank (PDB) (http://www.rcsb.org) database, while the structures of the candidate molecules, berberine (BBR), baicalein (BCL), luteolin (LTL), pravastatin, Metformin, and aspirin, were searched from the PubChem database (https://pubchem.ncbi.nlm.nih.gov). Then, the protein structure files were converted to *.pdbqt format and docked with drug molecules using AutoDock1.2.0 software by removing the original ligands and adding polar hydrogen atoms. AutoDock Vina software (ver. 1.1.2, the Scripps Research Institute, U.S.) was applied to derive binding free energies to simulate binding affinities between receptors and ligands. Finally, the folding patterns and molecular interactions between target proteins and drug molecules, as well as the bonds within the ligand-receptor complexes were visualized by Ligplot software (ver. 4.5.3, EBI, UK).

### Statistical analysis

Statistical analysis was performed via R software (version 4.0.3, http://www.r-project.org) and SPSS (version 27.0). Normally distributed continuous variables were recorded as the mean ± standard deviation (SD) and non-normal distributed variables were presented as median (range). Categorical variables are reported as numbers (proportions). Differences in continuous variables between two groups were calculated with student’s t-test or Mann-Whitney U-test. Differences in gene expression level were analyzed with the Wilcoxon rank-sum test and Kruskal-Wallis test. *P*<0.05 was considered a statistically significant difference.

## Results

### WGCNA construction based on autophagic gene set

**Fig. 1:**
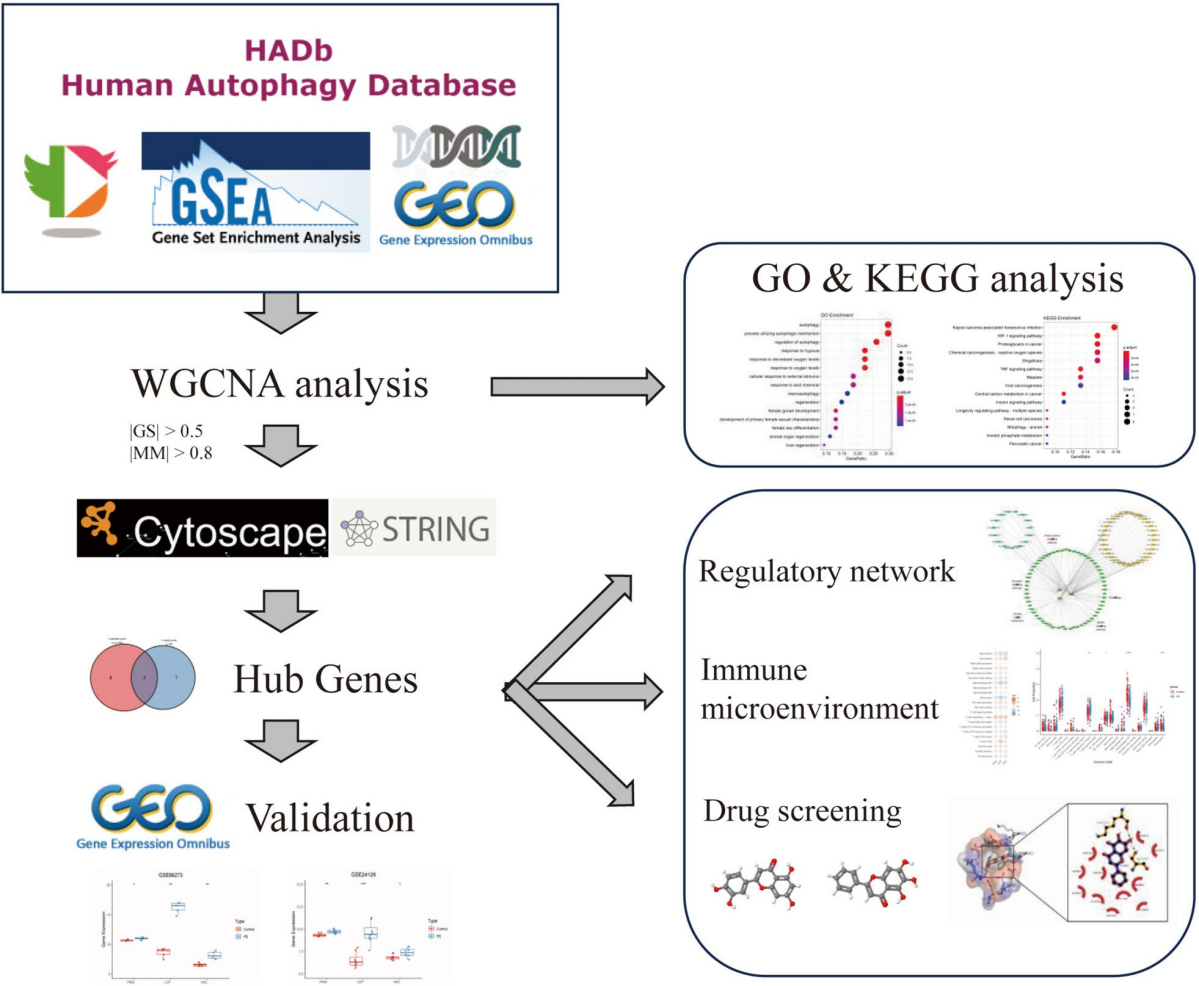
Flowchart of this study

**Fig. 2:**
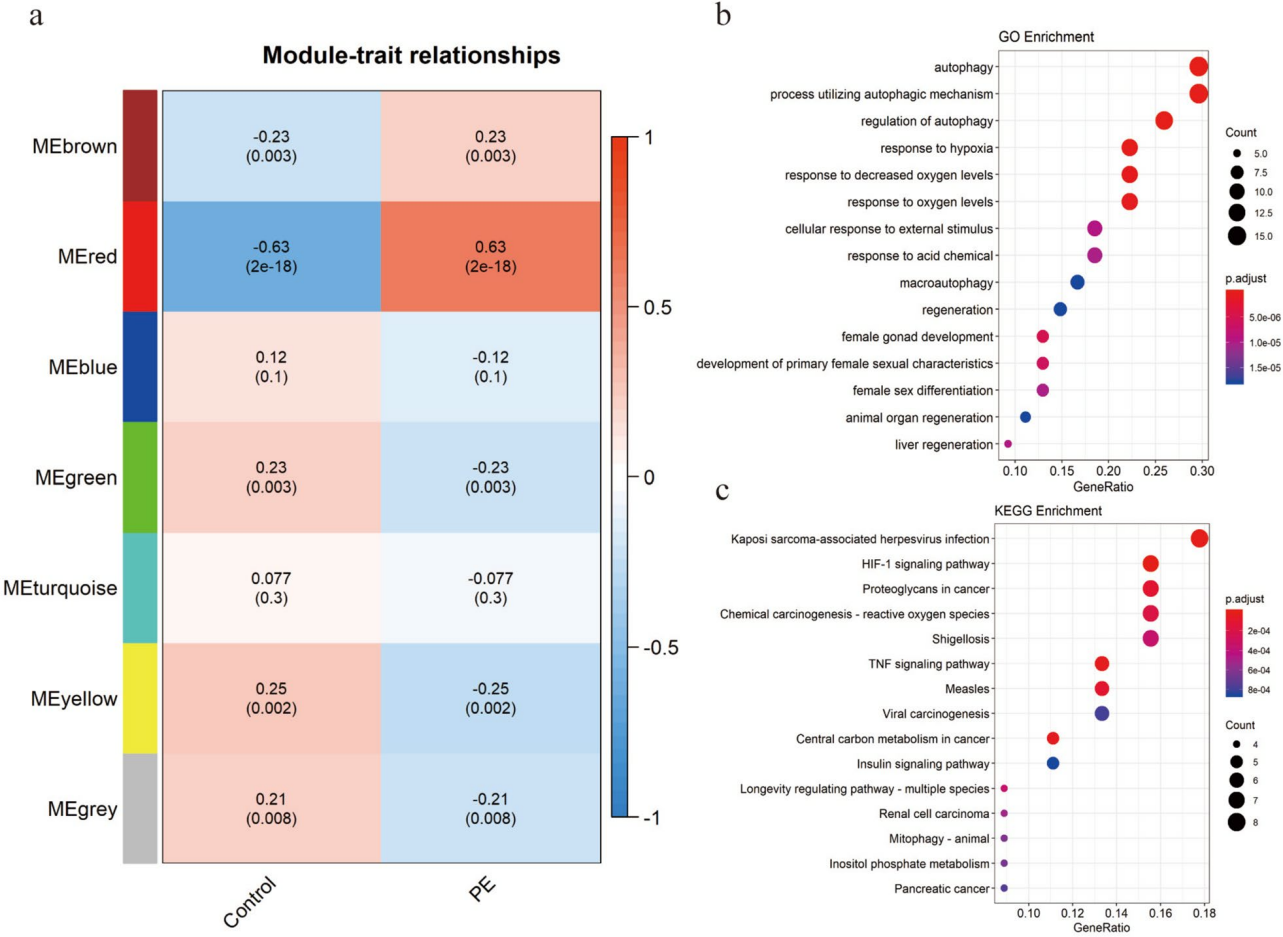
WGCNA analysis for autophagy-related genes that are associated with PE. **a** WGCNA analysis for gene modules. **b** GO functional enrichment analysis of genes in MEred. c) KEGG pathway analysis of genes in Mered, which were prepared with the KEGG database

The flowchart of this study can be found in Fig. 1. Clinical parameters of the PE group and non-PE group are presented in Table 1. A total of 7 modules were obtained in WGCNA analysis. The correlations between the sample trait and module eigengene (ME) of each module were calculated. As shown in Fig. 2a, MEred correlated most to PE, which included 54 genes. GO enrichment analysis of these genes indicated that autophagy, regulation of autophagy, response to hypoxia, and cellular response to external stimulus were the major bio-function of these genes (Supplementary file 3) (Fig. 2b). KEGG enrichment analysis suggested that HIF-1 signaling pathway and TNF signaling pathway were mainly involved (Supplementary file 4) (Fig. 2c).

### Hub genes screening and validation

**Fig. 3:**
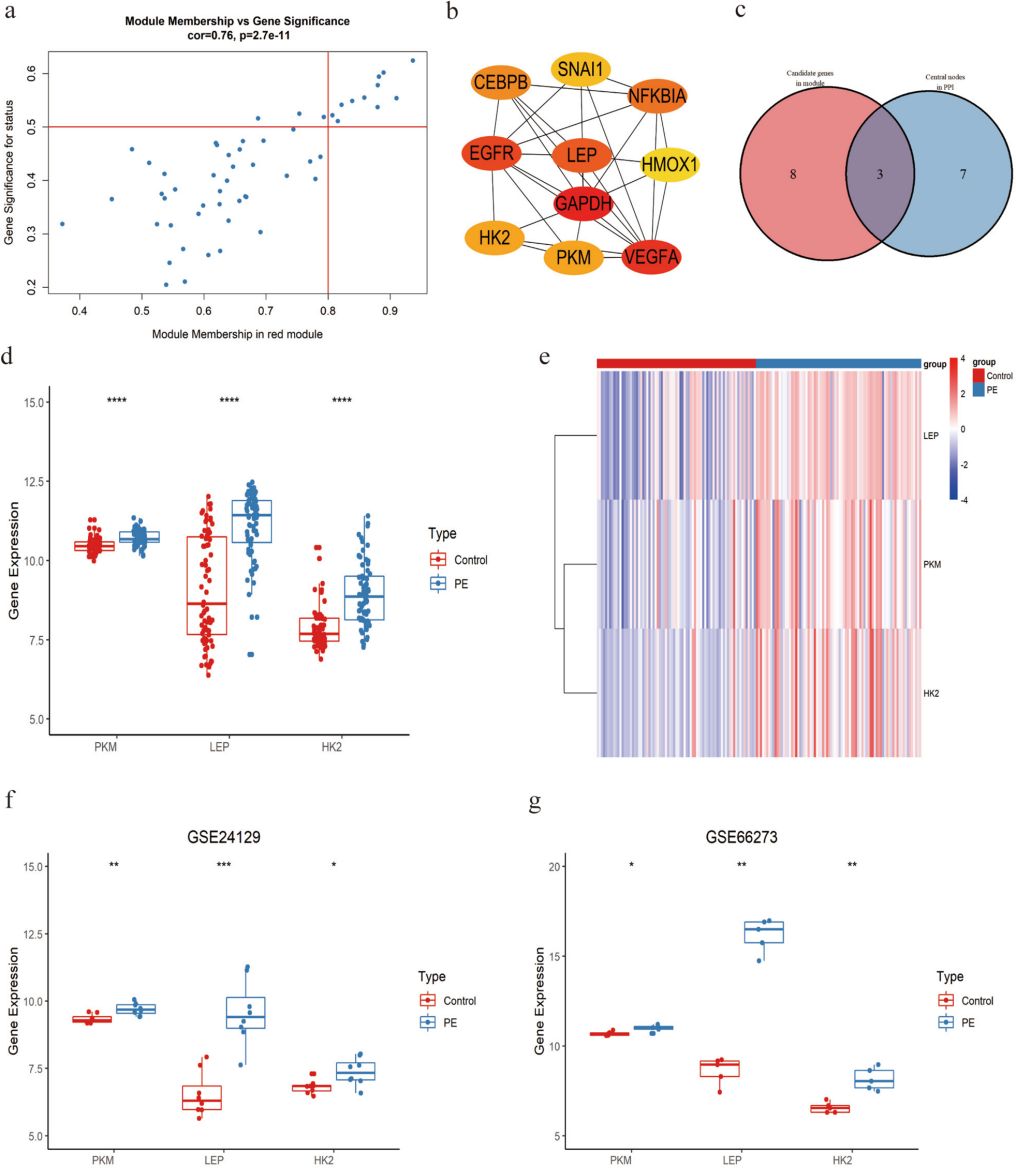
Hub genes screening and validation results. **a** Scatter plot of GS and MM values. **b** Top 10 genes filtered by cytohHubba and its correlations. **c** Venn graph of hub genes. **d** and **e** Expression of hub genes in the dataset GSE75010. **f** The expressions of PKM, LEP, and HK2 were higher in PE patients (*n*=8) than in the Control group (*n*=8) in dataset GSE24129 (*P*<0.01, *P*<0.001, *P*<0.05). **g**) The expressions of PKM, LEP, and HK2 were higher in PE patients (*n*=6) than in the Control group (*n*=5) in the dataset GSE66273 (*p<0.05, **p<0.01, ***p<0.001)

To filter the hub genes of autophagy-related PE differentially expressed genes (DEG), we obtained 11 module hub genes (Fig. 3a) and 10 PPI hub genes (Fig. 3b), respectively. Then, a total of 3 genes, namely LEP, PKM, and HK2, concurred in both gene sets, which were considered the hub genes (Fig. 3c). It showed that the hub genes were differentially expressed in embryo samples of the GSE75010 dataset (Fig. 3d, e). Besides, two independent datasets also showed that these genes were differentially expressed between PE patients and the control group (Fig. 3f, g).

### Correlations of hub genes and clinical parameters

**Fig. 4:**
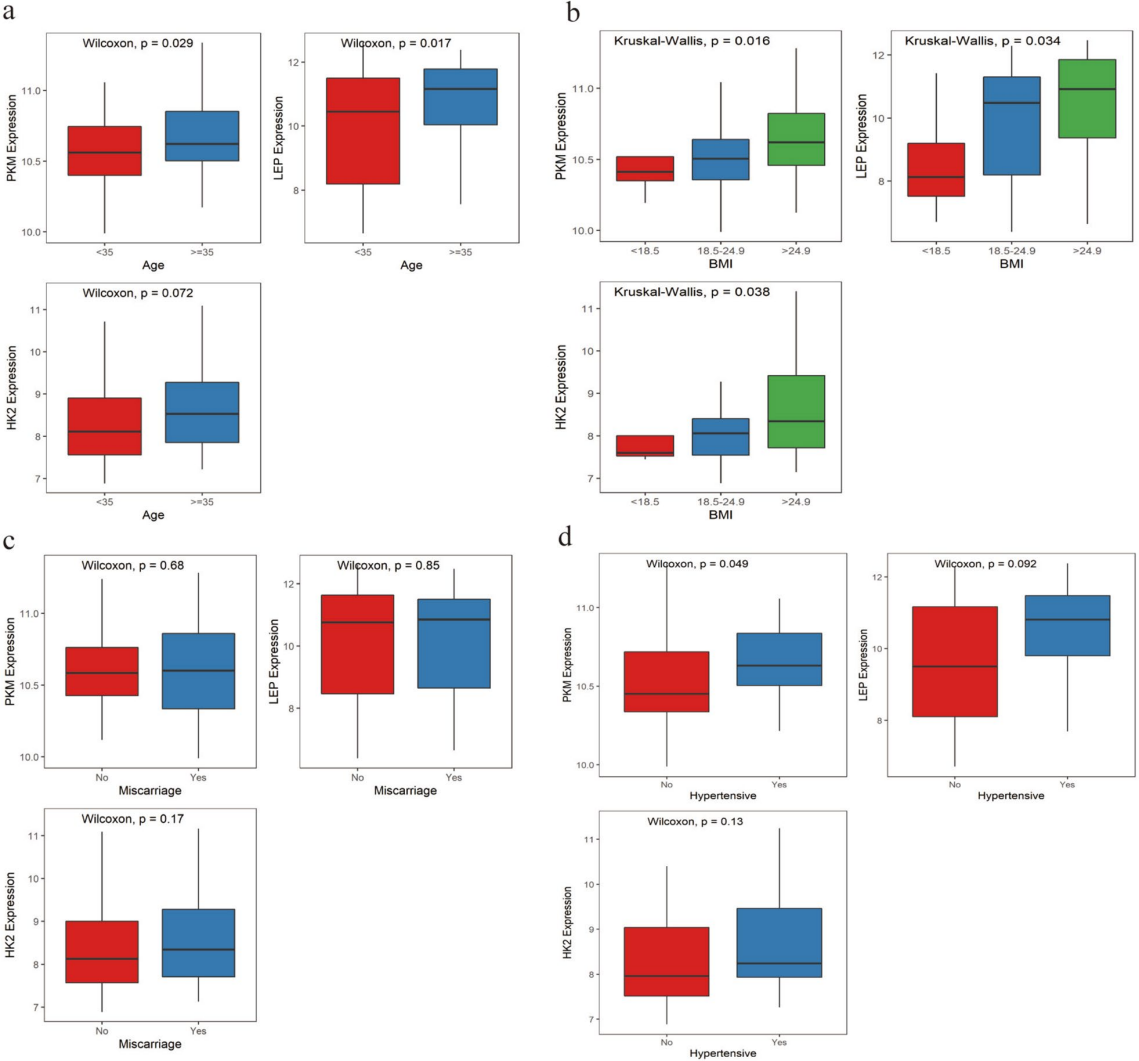
Differences in the expression of hub genes among subgroups. **a** Differences in the expression of hub genes between different age groups. **b** Differences in the expression of hub genes among BMI groups. **c** Differences in the expression of hub genes between different outcome groups. **d** Differences in the expression of hub genes among patients with or without hypertension

The expression levels of PKM and LEP were higher in patients older than 35 years old than that younger than 35-year-old (*p*<0.05; *p*<0.05) (Fig. 4a). Besides, there were significant differences among the three BMI groups in the expression of hub genes (all *p*<0.05) (Fig. 4b). However, the differences in the expression of hub genes between the miscarriage group and the non-miscarriage group, and between the hypertensive group and non-hypertensive group were small, except that the expression of PKM was remarkably higher in the hypertensive group than that in the non-hypertensive group (*p*<0.05) (Fig. 4c, d).

### Potential molecular mechanisms of hub genes

**Fig. 5:**
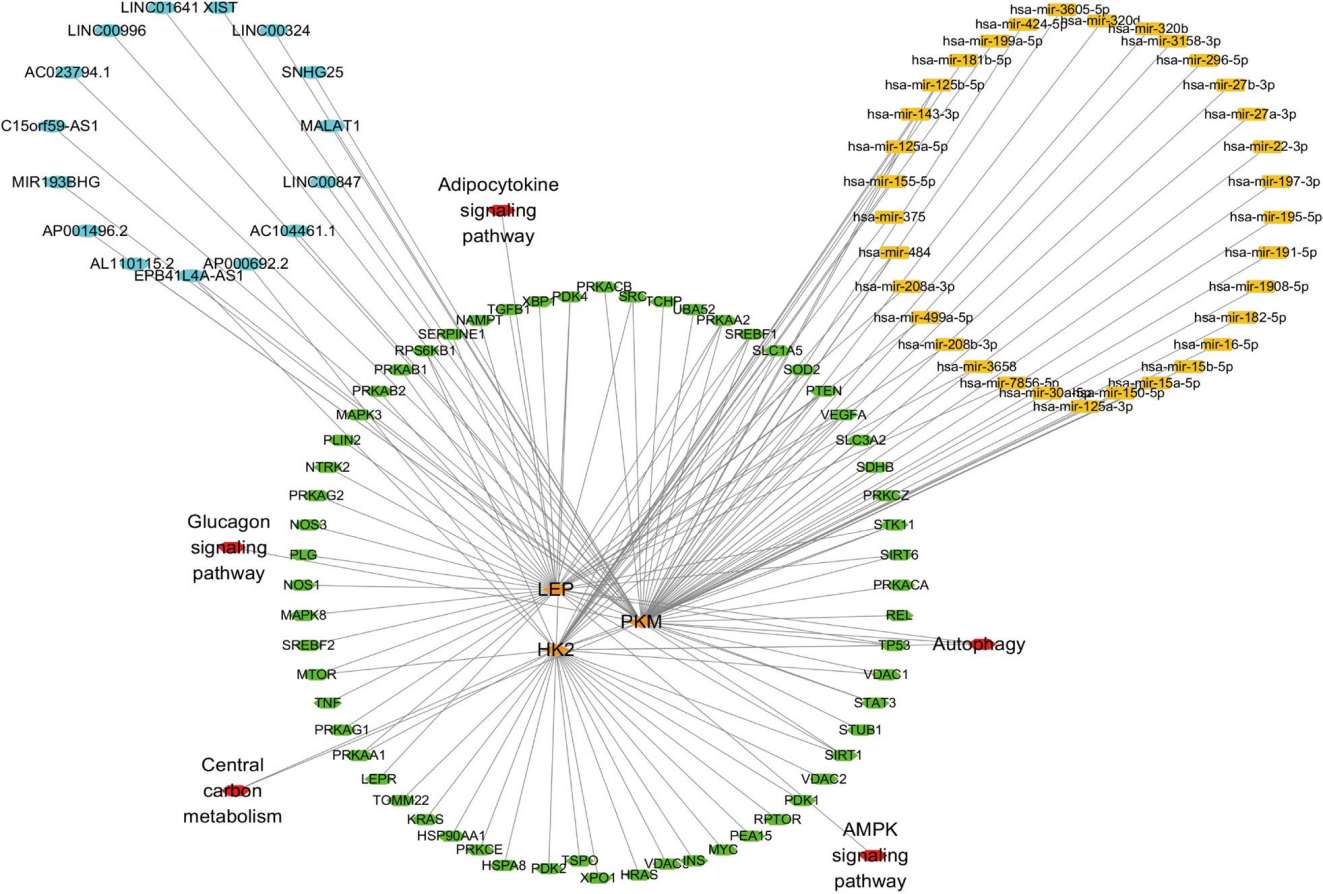
Multifactor interaction network of hub genes, autophagic genes, miRNA, lncRNA, and signaling pathways. Orange ovals referred to hub genes; green ovals referred to autophagic genes; blue ovals referred to lncRNA; yellow marks represented miRNA; red ovals stood for signaling pathways

A multifactor network was constructed which included 3 hub genes, 61 autophagic genes, 15 lncRNAs, 92 miRNAs, and 5 signaling pathways (Fig. 5). PKM interacted with 22 autophagic genes, 81 miRNAs, and 14 lncRNA, and was involved in Autophagy, Central carbon metabolism, and Glucagon signaling pathways. LEP interacted with 32 autophagic genes and 5 miRNAs and was involved in Autophagy, AMPK signaling pathway, and Adipocytokine signaling pathway. Meanwhile, HK2 interacted with 31 autophagic genes, 9 miRNAs, and 2 lncRNA, and was involved in Autophagy and Central carbon metabolism.

### Association between hub genes and immune microenvironment

**Fig. 6:**
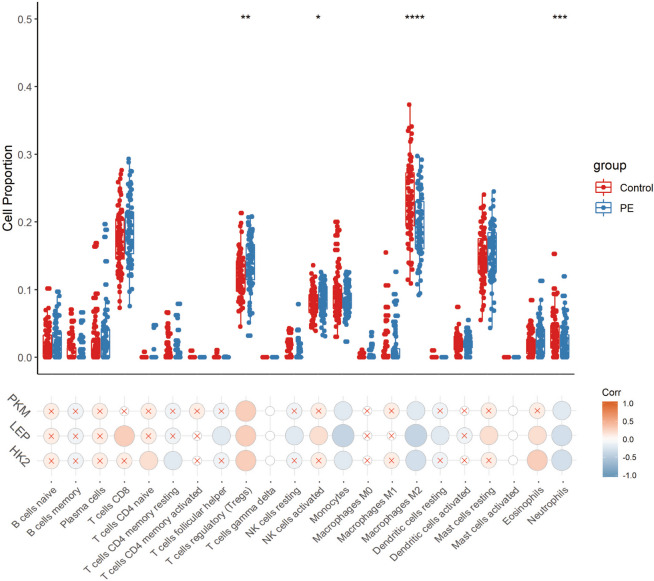
The abundance of infiltrated immune cells in PE placenta samples and correlations between hub gene expression levels and immune cell proportions

As shown in Fig. 6, the proportions of Tregs (*p*<0.01), activated NK cells (*p*<0.05), M2 macrophages (*p*<0.0001), and neutrophils (*p*<0.001) differed remarkably between the PE group and non-PE group. We further calculated the correlations between hub gene expression level and immune cell proportions via Pearson analysis and found that Tregs were positively related to PKM (*r*=0.3, *p*<0.001), LEP (*r*=0.3, *p*<0.0001), and HK2 (*r*=0.3, *p*<0.001), while M2 macrophages were negatively related to PKM (*r*=-0.2, *p*<0.05), LEP (*r*=-0.4, *p*<0.0001), and HK2 (*r*=-0.3, *p*<0.001).

### Molecular docking results

**Fig. 7:**
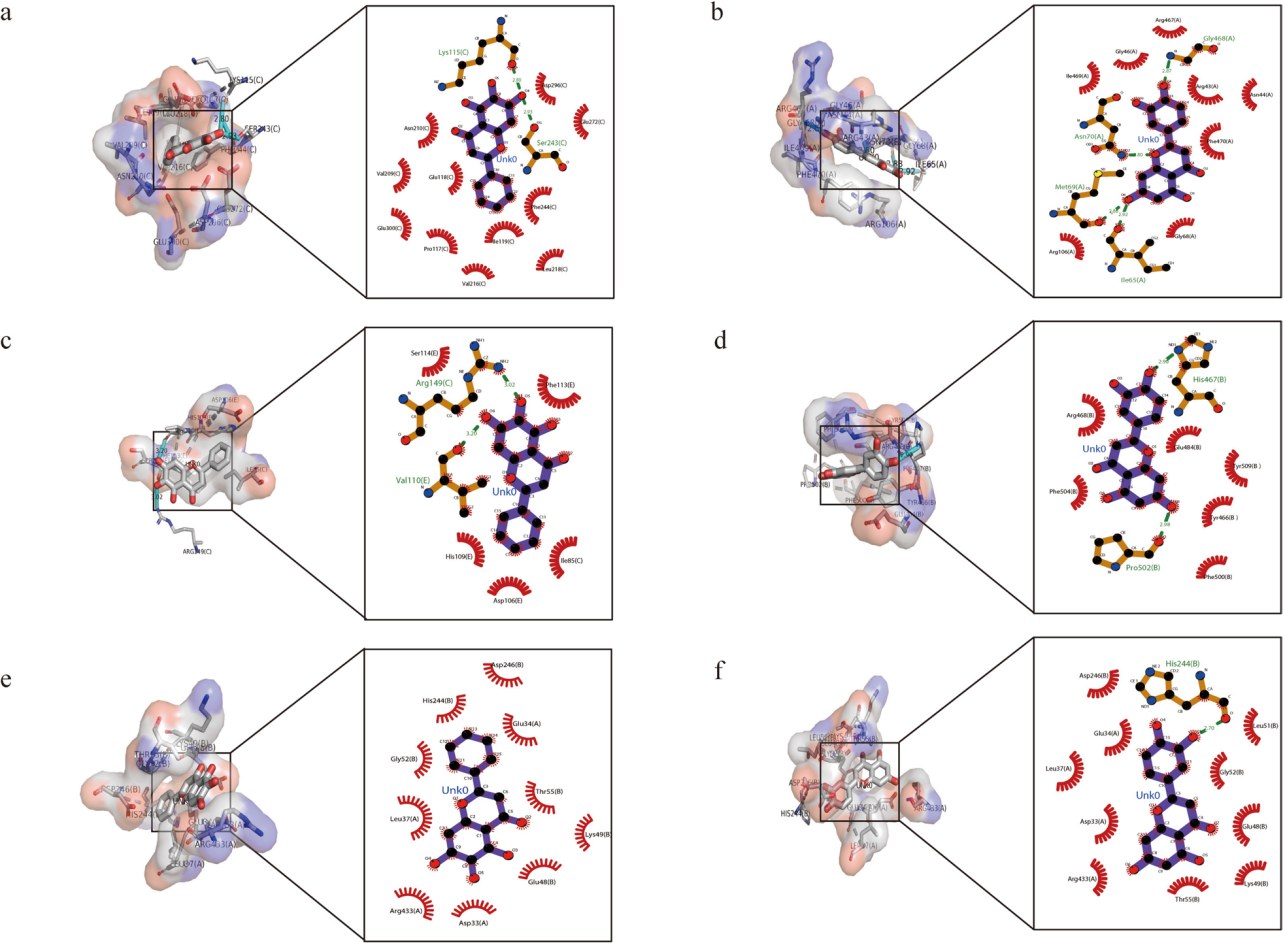
Molecular docking results of candidate molecules and target proteins. Interactions of baicalein (**a**) and luteolin (**b**) binding to PKM2. Interactions of baicalein (**c**) and luteolin (**d**) binding to leptin. Interactions of baicalein (**e**) and luteolin (**f**) binding to Hexokinase 2

The binding free energies of aspirin, metformin, pravastatin, and berberine to the target proteins were relatively low (>-7.5 kcal/mol) when compared to that of baicalein and luteolin (Table 2). As shown in Fig. 7, baicalein has the highest affinity to PKM (-8.5 kcal/mol), with 2 hydrogen bonds and 11 residues involved in hydrophobic contacts, while luteolin has the highest affinity to HK2 (-8.9 kcal/mol), with 1 hydrogen bond and 10 residues involved in hydrophobic contacts.

**Table 2:** Affinities of candidate molecules binding to target proteins

Candidate molecule	Target Protein ID	Binding affinity (kcal/mol)	Hydrophobic contact^a^	Hydrogen bond and its length
Aspirin	PKM_6ttf	-6.5	4	Arg106(3.02); Arg43(2.83); Asn70(3.09)
	LEP_7z3q	-5.1	8	/
	HK2_5HFU	-5.5	6	Glu48(2.80)
Metformin	PKM_6ttf	-5.4	2	Asp113(3.05); Thr114(2.95); Lys115(3.15); Ser243(3.15); Lys270(2.96); Glu272(3.16); Asp296(3.19)
	LEP_7z3q	-4.8	/	Thr33(2.96); Asn567(3.16); Asn566(3.04); Asp617(3.10)
	HK2_5HFU	-5.3	3	Arg462(3.08); Ser70(2.94); Thr71(3.07); Pro72(3.04)
Pravastatin	PKM_6ttf	-6.5	8	Asn210(2.91); Ser243(3.16); Lys270(3.21); Arg73(2.83); Asn75(3.05); Ser77(3.10)
	LEP_7z3q	-6.3	12	Leu471(2.90)
	HK2_5HFU	-6.7	6	Arg373(3.14); Arg426(2.97); Arg433(3.11); Asp33(2.94); Leu31(2.94)
Berberine	PKM_6ttf	-6.5	4	Arg447(3.12); Thr41(2.94)
	LEP_7z3q	-6.6	7	Thr33(3.28); Asn566(3.06)
	HK2_5HFU	-7.2	7	Lys418(3.21)
Baicalein	PKM_6ttf	-8.5	11	Lys115(2.80); Ser243(2.93)
	LEP_7z3q	-7.7	5	Arg149(3.02); Val110(3.20)
	HK2_5HFU	-8.0	10	/
Luteolin	PKM_6ttf	-8.4	8	Gly468(2.87); Asn70(2.80); Met69(2.88); Ile65(2.92)
	LEP_7z3q	-7.3	6	Pro502(2.98); His467(2.90)
	HK2_5HFU	-8.9	10	His244(2.70)

^a^Refers to number of residues involved in hydrophobic contacts

## Discussion

In this study, we applied a WGCNA analysis based on public databases and performed GO and KEGG enrichment analysis for the most correlated module. It turned out that hypoxia and oxidative stress were the major bioprocesses in PE, which is consistent with our prior knowledge. After further screening via the String database, we found 3 autophagy-related hub genes overexpressed in the PE group, namely, PKM, LEP, and HK2. In two independent datasets, these hub genes were also upregulated in the PE group. We further analyzed the hub gene expression among different subgroups and found that PKM and LEP were remarkably upregulated in women over 35 years old than those younger than 35 years old. Besides, women with a higher BMI tended to present higher expression of hub genes, and women with hypertension had a higher expression of PKM compared to women without. These results indicated that aging and energy metabolism were associated with autophagy. PKM encodes a pyruvate kinase involved in glycolysis, which has four isoforms (M1, M2, L, and R) in mammalian cells. Among these isoforms, PKM1 is a constitutively active enzyme in normal adult cells [41], while PKM2 is conditionally active and predominantly expressed in fetus and cancer cells [42]. Contrary to PKM1, the low glycolysis activity of PKM2 can limit glucose oxidation and allow cells to grow under hypoxia [43, 44]. This may explain the high expression of PKM (PKM2) in the placentas of PE group as a result of adaptation to the hypoxia environment. Therefore, activation of PKM2 might alter the low flux and improve energy depletion caused by hypoxia. LEP is located on chromosome 7, which encodes a hormone peptide leptin that plays a capital role in energy homeostasis. Except for white adipocytes, leptin can be also produced by other tissues, including endometrium, placenta, and umbilical cord, with a wide range of functions in regulation of angiogenesis, vascular function, blood pressure, immunity, and placenta development [45–47]. Studies reported that hypoxia could lead to placenta leptin gene expression and production [48], and abnormal placenta leptin level was associated with aberrant trophoblast proliferation or invasion, as well as endothelial dysfunction, hypertension and fetal growth restriction [49, 50], which may lead to PE. The interacting relations between leptin and autophagy varies in a tissue-specific pattern [51]. Although, the evidence in placenta is scarce, it was reported that autophagy was active in the placenta of PE women and leptin could modulate autophagy in various tissue types [52–55]. HK2 is a protein-encoding gene that encodes hexokinase 2, an enzyme that catalyzes glucose into phosphorylation form. It was suggested that HK2 was pivotal to decidualization and non-coding RNA of HK2, including lncRNA and miRNA, could lead to dysfunction of HK2 and possibly cause PE [56]. Therefore, we further investigated the regulation network among hub genes, lncRNA and miRNA to explore the underlying mechanisms of hub genes in the pathogenesis of PE and found that the signaling pathway involved were mainly autophagy and other metabolic pathways. These results indicated that autophagy might mediate PE via metabolism disorder.

In addition to the placenta metabolic disorders, maternal factors may also be involved, such as maternal immune function. It is well established that immune-inflammation disorder at the maternal-fetus interface participates in PE development. For example, natural killer (NK) cells and Tregs in the decidua influence placentation; inhibition of NK response to MHC and deficient in Tregs may lead to impaired spiral artery remodeling [57, 58]. However, our studies showed an elevated proportion of activated NK cells and Tregs in PE group. This discrepancy might be result of mRNA-based analysis of this study, which only reflected quantities but lacked functional information of these immune cells. Macrophages obsess potential for phenotypic plasticity, which are featured by immunosuppressive M2 phenotype and inflammatory M1 phenotype. During normal pregnancy, M2 macrophages predominant in decidua to support fetus-maternal immune tolerance. Whilst in hypertensive pregnancy, aberrant macrophage activation may lead to insufficient trophoblast invasion and induce placenta apoptosis [59]. In this study, the M1 macrophages in PE group was numerically higher than healthy women, but the difference was small; while M2 macrophages were remarkably lower in PE, indicating the imbalance of macrophages in PE placentas. Yang et al. [60] reported spontaneous miscarriage women displayed inhibited autophagy and reduced retention of decidua macrophages, which was mediated by a lipid metabolite. This study suggested that metabolism reprogramming can regulate immune balance via autophagy and subsequently result in poor outcomes in pregnant women. Multiple studies have mentioned the immune-regulatory roles of the hub genes. For example, PKM2 was found to promote M1 type activation of macrophages [61], which might explain the upregulated PKM and downregulated M2 macrophages in PE group. Besides, PKM2 could stimulate activation of NK and neutrophils [62, 63], possibly leading to an inflammatory environment for PE pathogenesis. Nevertheless, these results were based on other tissue types. In the future, more studies should be performed on PE population to investigate the relationship between hub genes and immune cells in placenta.

Contemporary research has focused on inventive therapy for PE, including aspirin, statins, metformin, and other recombinant proteins. Aspirin is a non-steroid anti-inflammatory agent that also reduces sFlt-1 overexpression to counteract PE [64]. Similarly, statins and metformin were shown to reverse placental perfusion by sFlt-1 inhibition [65, 66]. In this study, however, these small molecules didn’t display high affinities to autophagy-related hub genes according to the molecular docking results. Molecular docking is an essential tool for drug design with its ability to predict the conformation of small molecules and targets [67], and it has been applied in many studies to screen potential drugs [68, 69]. The docking results showed there was a low binding affinity of BBR to hub genes, indicating autophagy might not be involved in the working mechanisms of BBR when used in PE. In contrast, BCL and LTL displayed high affinities to hub genes with binding affinity being as high as -8.4 and -8.9, respectively, which means they may bind directly to the hub genes of PE and take effect. What’s more, numerous research suggested BCL and LTL could modulate autophagy in various tissue types [70–73]. For example, BCL can promote autophagy and reduce ROS in cardiac muscle [72], while luteolin can inhibit autophagy via mTOR signaling in lung tissues [70]. Although the research rendered inconsistent results, which might be due to different tissues and diseases, at least they displayed a potential of BCL and LTC to target autophagy. Taken together, we speculate BCL and LTL might be the promising drugs that target autophagy in PE, but more solid proof should be provided in the future.

Limitations inevitably exist in this study. First, the results were based on public datasets which lack detailed clinical information about pregnant women. Second, the mode of action of hub genes and specific bioeffects of potential drugs after binding to hub genes remain elusive. More functional experiments and mechanism research should be performed to verify the results.

In summary, this research has identified PKM, LEP, and HK2 to be promising biomarkers for preeclampsia, which might regulate the pathogenesis of preeclampsia via targeting autophagy, metabolism and immune microenvironment. Molecular docking simulation predicted baicalein and luteolin to be promising novel drugs for PE. Hopefully, this study might highlight the role of autophagy in PE and contribute to the drug development for PE.

### Supplementary Information

**Additional file 1: Supplementary file 1.** List of autophagy-related genes.

**Additional file 2: Supplementary file 2.** The signature matrix file of LM22.

**Additional file 3: Supplementary file 3.** GO enrichment analysis result.

**Additional file 4: Supplementary file 4.** KEGG enrichment analysis result.

## Data Availability

The authors confirm that the data supporting the findings of this study are available within the article and its supplementary materials.

## Abbreviations

PE: Preeclampsia
HELLP: Hemolysis, elevated liver enzymes and low platelet count syndrome
BBR: Berberine
BCL: Baicalein
WGCNA: weighted gene co-expression network analysis
GEO: Gene Expression Omnibus
KEGG: Kyoto Encyclopedia of Genes and Geno
GO: Gene Ontology
BP: Biological process
MF: Molecular function
CC: Cellular component
GS: Gene significance
MM: Module membership
PPI: Protein-protein interacting network
TF: Transcription factors
PDB: Protein Data Bank
LTL: Luteolin
SD: Standard deviation
ME: Module eigengene
DEG: Differentially expressed genes
AMPK: Adenosine 5’-monophosphate (AMP)-activated protein kinase
NK: Natural killer
ROS: Reactive oxygen species
BMI: Body mass index
